# A Novel Volume-Stable Collagen Matrix Induces Changes in the Behavior of Primary Human Oral Fibroblasts, Periodontal Ligament, and Endothelial Cells

**DOI:** 10.3390/ijms22084051

**Published:** 2021-04-14

**Authors:** Maria B. Asparuhova, Alexandra Stähli, Kevin Guldener, Anton Sculean

**Affiliations:** 1 Dental Research Center, Laboratory of Oral Cell Biology, School of Dental Medicine, University of Bern, 3010 Bern, Switzerland; 2 Department of Periodontology, School of Dental Medicine, University of Bern, 3010 Bern, Switzerland; alexandra.staehli@zmk.unibe.ch (A.S.); kevinguldener@bluewin.ch (K.G.); anton.sculean@zmk.unibe.ch (A.S.)

**Keywords:** wound healing, hemostasis, fibrin clot, fibrinolysis, growth factors, connective tissue grafts, xenografts, biomaterials, soft tissue regeneration, transcription

## Abstract

The aim of the present study was to investigate the influence of a novel volume-stable collagen matrix (vCM) on early wound healing events including cellular migration and adhesion, protein adsorption and release, and the dynamics of the hemostatic system. For this purpose, we utilized transwell migration and crystal violet adhesion assays, ELISAs for quantification of adsorbed and released from the matrix growth factors, and qRT-PCR for quantification of gene expression in cells grown on the matrix. Our results demonstrated that primary human oral fibroblasts, periodontal ligament, and endothelial cells exhibited increased migration toward vCM compared to control cells that migrated in the absence of the matrix. Cellular adhesive properties on vCM were significantly increased compared to controls. Growth factors TGF-β1, PDGF-BB, FGF-2, and GDF-5 were adsorbed on vCM with great efficiency and continuously delivered in the medium after an initial burst release within hours. We observed statistically significant upregulation of genes encoding the antifibrinolytic thrombomodulin, plasminogen activator inhibitor type 1, thrombospondin 1, and thromboplastin, as well as strong downregulation of genes encoding the profibrinolytic tissue plasminogen activator, urokinase-type plasminogen activator, its receptor, and the matrix metalloproteinase 14 in cells grown on vCM. As a general trend, the stimulatory effect of the vCM on the expression of antifibrinolytic genes was synergistically enhanced by TGF-β1, PDGF-BB, or FGF-2, whereas the strong inhibitory effect of the vCM on the expression of profibrinolytic genes was reversed by PDGF-BB, FGF-2, or GDF-5. Taken together, our data strongly support the effect of the novel vCM on fibrin clot stabilization and coagulation/fibrinolysis equilibrium, thus facilitating progression to the next stages of the soft tissue healing process.

## 1. Introduction

Various regenerative surgical techniques and biomaterials have been utilized in the oral cavity aiming to obtain predictable coverage of single and multiple gingival recessions at natural teeth and gain of attached mucosa at dental implants [1]. Autogenous palatal subepithelial connective tissue grafts (CTGs) are still considered the gold standard in enhancing the soft tissue regeneration in the oral cavity, and have shown excellent clinical outcomes in most indications, e.g., for attaining complete root coverage [2]. However, autogenous tissue harvesting significantly increases patient morbidity through the generation of a second surgical site, thus prolonging the time of the surgical procedure and increasing postoperative discomfort and risk of complications [3,4,5]. Furthermore, patient-specific anatomical features such as thin gingival phenotype or shallow palate appear determinant for the quality and quantity of the harvested autologous tissue [3,4]. Soft tissue substitute materials of different origins, e.g., allogenic dermal substitutes or xenogenic collagen matrices, have been introduced in periodontal and peri-implant plastic surgeries as an alternative to autografts [6]. Due to the lack of a cellular component and the preservation of their mechanical stability by chemical modifications (crosslinking), these collagen-based materials serve as safe three-dimensional scaffolds developing into a fully functional tissue by enabling repopulation of different oral cell types and in-growth of blood vessels and epithelium from the surrounding tissues.

In agreement with the inherited ability of collagen to bind a number of proteins [7], we recently showed that the transforming growth factor-β1 (TGF-β1), fibroblast growth factor-2 (FGF-2), platelet-derived growth factor-BB (PDGF-BB), growth and differentiation factor-5 (GDF-5), or the bone morphogenetic protein-2 (BMP-2) can be adsorbed to commercially available porcine-derived collagen matrices with an efficiency close to 100%, and are then continuously released over time [8]. Furthermore, the four investigated collagen scaffolds have provided a favorable environment able to successfully promote the migration, adhesion, and proliferation of two oral cell types, namely primary human oral fibroblasts (hOF) and periodontal ligament cells (hPDLC) [9]. A number of clinical studies have demonstrated the successful application of acellular collagen matrices in periodontal surgical reconstructions, with low patient morbidity [10,11,12,13,14,15]. Although collagen matrices are considered the graft substitutes providing the most similar outcomes to the autogenous CTGs, the majority of clinical studies comparing the two graft types have demonstrated the superiority of the CTGs, particularly in the treatment of gingival recessions [2,5,16,17,18] as well as in soft tissue augmentation procedures for increasing the width of keratinized tissue [15,19]. Thus, a comparison of an acellular dermal allograft with an autogenous CTG, both combined with coronally advanced flap in the treatment of surgically created Miller Class I recession defects, demonstrated a significantly higher gingival thickness in the CTG group, in spite of a similar graph thickness (1 mm) at the time of the surgical placement [20]. Furthermore, in a randomized clinical trial, Pietruska and colleagues showed that subepithelial CTG excels a collagen matrix in decreasing the gingival recession height and width, increasing the gingival thickness and the formation of keratinized tissue [5]. Compared to CTGs, collagen-based matrices appear to be more prone to contraction, explaining the observed reduction in the tissue thickness [15,19].

To overcome the volume stability limitation of most commercially available collagen-based substitutes, a novel, highly porous and volume-stable collagen matrix (vCM) was recently introduced for soft tissue augmentation around teeth and dental implants [21]. This vCM of porcine origin is predominantly made of collagen type I and III and a small portion of elastin. vCM consists of a single porous layer with interconnected pores (93% volume porosity) and an average pore size of 92 µm. While mechanical stability is achieved by chemical crosslinking, mechanical testing demonstrated preserved elasticity of the material over 14 days [22]. Preclinical and clinical studies have demonstrated excellent biocompatibility and favorable outcomes in soft tissue augmentation using vCM, with a minimal loss of tissue thickness six months after implantation [21,23,24,25,26,27,28]. Recent histological data showed that the biomaterial adsorbed blood after its implantation without collapsing [26,28]. Moreover, the matrix maintained sufficient volume stability to allow fast ingrowth of blood vessels and vimentin-positive mesenchymal cells producing collagen type I, resulting in complete integration in the host tissue before its degradation occurred [26,27].

The formation of fibrin clot is an early event in the wound healing process [29]. The fibrin clot not only prevents from blood loss by sealing the tissue after wounding, but also provides adhesion for leucocytes, macrophages, fibroblasts, and endothelial cells. Furthermore, it serves as a reservoir for autogenous growth factors that stimulate the subsequent stages of the soft tissue healing [30]. The dynamics of the hemostatic system is exceedingly complex and involves a fine-tuned balance between coagulation and fibrinolysis [31]. The regulation of the two processes is achieved by the activity of a number of anti- and pro- fibrinolytic factors. Among them are thrombomodulin (encoded by the THBD gene) [31], thrombospondin 1 (encoded by THBS1) [32,33,34], thromboplastin, also known as tissue factor (encoded by F3) [35], plasminogen activator inhibitor type 1 (encoded by SERPINE1) [36], tissue plasminogen activator (encoded by PLAT) [31], urokinase-type plasminogen activator (encoded by PLAU) and its receptor (encoded by the PLAUR) [31], and the matrix metalloproteinase 14 (encoded by MMP14) [37]. Most of these factors also perform functions in cellular motility, adhesion, and differentiation [38]. Impairment of the control over the hemostatic dynamics leads to a number of serious physiological complications. Increased fibrinolysis, possibly related to clot instability, is noted in patients after surgery or major trauma, in cirrhosis, renal failure, menorrhagia, and some malignancies [39]. In contrast, decreased fibrinolysis relates to the development of thrombosis and is seen in atherosclerosis, diabetes, and obesity [40]. The capacity of a biomaterial developed for soft tissue augmentation to maintain a balanced hemostatic system, namely, to stabilize the fibrin clot and prevent flap collapse as well as to offer an environment that will not impair the dissolution of the clot once it has served its purpose in hemostasis, is an important prerequisite for successful tissue regeneration to occur.

Knowledge about the influence of vCM on the early wound healing events, including the dynamics of the hemostatic system, cell migration, and adhesion to the matrix, is currently missing. Furthermore, the combination of vCM with growth and differentiation factors is underinvestigated. Therefore, the aim of the present laboratory-based study was to investigate the behavior of primary hOF, hPDLC, and human umbilical vein endothelial cells (HUVEC) to vCM in terms of cellular migration and adhesion to the matrix. Each of these cell types represent a well-accepted model for studying the responses of oral (hOF and hPDLC) and endothelial (HUVEC) cells to various stimuli and regenerative biomaterials [41,42,43]. Furthermore, we quantitatively investigated the adsorption capacity of the vCM for growth factors typically present in the wound environment as well as the subsequent growth factor release over a 12-day period. Finally, the expression of genes involved in the regulation of the fibrinolytic process was quantitatively determined in each of the above cell types cultured either on native/uncoated or growth factor-coated vCM. That way, we also answered the question of how preserved and/or modulated the functional activity of the growth factors was after being released from the matrices.

## 2. Results

### 2.1. Strongly Increased Migratory Potential of Primary hPDLCs, hOFs, and HUVECs toward the Volume-Stable Collagen Matrix

**Figure 1:**
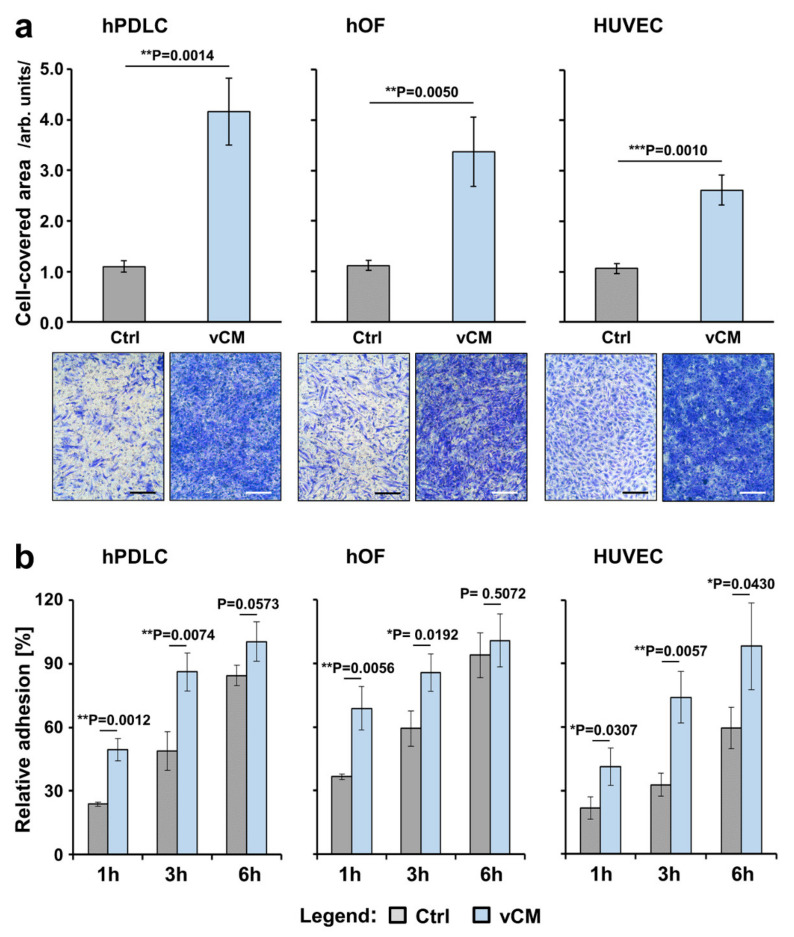
Promigratory (**a**) and proadhesive (**b**) effects of vCM on three primary cell types of human origin. (**a**) Migratory capacity of hPDLCs (left panel), hOFs (middle panel), and HUVECs (right panel) toward vCM was examined by modified Boyden chamber assay using cell culture inserts with permeable membranes (8 µm pore size). Following an incubation period of 18 h, cells that had migrated through the membrane were stained and quantified by using ImageJ. Cells that migrated in the absence of vCM served as a control (Ctrl). Means ± SD from three independent experiments performed in duplicates and a significant difference between the control and test group, ** *p* < 0.01, *** *p* < 0.001 are shown; Representative images of crystal violet-stained cells in each of the experimental groups are shown below the bar charts. Scale bar, 500 μm. (**b**) Adhesive capacity of hPDLCs (left panel), hOFs (middle panel), and HUVECs (right panel) on vCM was examined using crystal violet staining of adherent cells at 1, 3, and 6 h postseeding. Controls (Ctrl) represent cells of each cell type seeded on tissue culture-treated plates in the absence of vCM for the indicated times. Normalization of the experimental values to the values obtained for the total number of seeded control cells (taken as 100% adhesion) was applied. Means ± SD from three independent experiments performed in duplicates and significant differences to control cells at each time point, ** *p* < 0.01, * *p* < 0.05 are shown.

The potential of primary hPDLCs, hOFs, and HUVECs to migrate toward the commercially available vCM was investigated by using transwell permeable supports in a modified Boyden chamber migration assay. The primary hPDLCs and hOFs exhibited 3.0- and 3.8-fold greater migration, respectively, toward vCM compared to control cells that migrated in the absence of a matrix (*p* < 0.01; Figure 1a). Compared to the basal migration of control cells, the migratory capacity of HUVECs toward vCM was increased by 2.5-fold with a great statistical significance (*p* = 0.001; Figure 1a).

### 2.2. Significantly Increased Adhesive Properties of Primary hPDLCs, hOFs, and HUVECs on the Volume-Stable Collagen Matrix

We next investigated the adhesive properties of the primary hPDLCs, hOFs, and HUVECs cultured on tissue culture plastic (control group) or vCM (test group) over 1, 3, and 6 h. The adhesive potential of each of the three cell types increased continuously over the entire test period, reaching > 85% adhesion at 6 h in both control and test group for hPDLCs and hOFs, and reaching 98% in the test group versus only 60% in the control group for HUVECs (Figure 1b). In primary hPDLCs, significantly higher adhesion of 50 and 86% was detected in the test group versus 24 and 49% in the control group at 1 and 3 h, respectively (compare gray with blue bars, *p* < 0.01). Similarly to the hPDLCs, 69 and 86% adhesion of hOFs on vCM was detected 1 and 3 h postseeding, respectively, compared to 37 and 59% in the control group (*p* < 0.05). In contrast to hPDLCs and hOFs, the relative adhesion rates of HUVECs cultured on vCM remained significantly higher (*p* < 0.05) than the control at each time point tested.

### 2.3. Adsorption and Release of Recombinant Growth Factors from the Volume-Stable Collagen Matrix over a 12-Day Period

**Figure 2:**
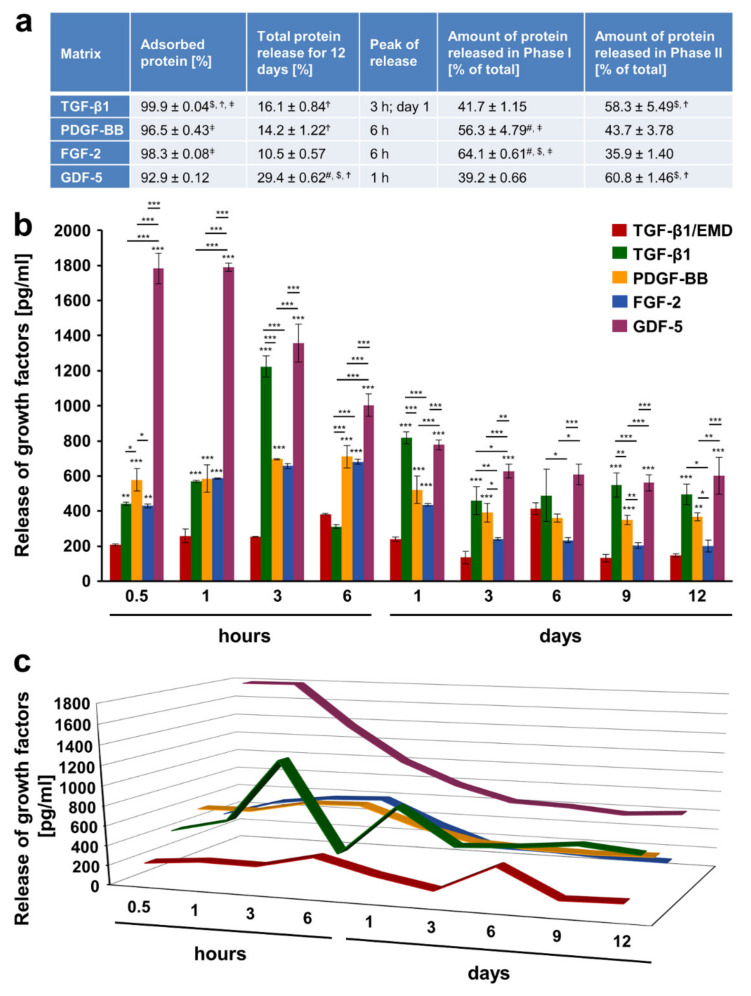
Adsorption and release of various recombinant growth factors from vCM. The vCM was incubated for 10 min at room temperature in phosphate-buffered saline (PBS) containing 100 ng/mL of TGF-β1, PDGF-BB, FGF-2, or GDF-5. (**a**) Colorimetric ELISAs were performed for: (1) quantitative evaluation of adsorption rate (expressed in percent) of each of the four growth factors; (2) quantitative evaluation of total protein release (expressed as percent of adsorbed protein) from vCM for a 12-day period for each of the growth factors; (3) determination of the peak of release, i.e., the time point at which the highest growth factor release from the vCM coated with the respective growth factor was observed; (4) quantitative evaluation of growth factor release in phase I expressed as percent of the total release for the entire 12-day period (taken as 100%); (5) quantitative evaluation of growth factor release in phase II expressed as in (4). The peak release marks the end of phase I whereas phase II spans the period immediately after the peak of the release until day 12. Means ± SD from three independent experiments and significant differences (p < 0.05) between the experimental groups are shown. Significance was indicated with the following symbols: # denotes significantly higher than TGF-β1, $ denotes significantly higher than PDGF-BB, Ϯ denotes significantly higher than FGF-2, and ǂ denotes significantly higher than GDF-5. (**b**) Release of TGF-β1 from vCM coated with EMD as well as release of TGF-β1, PDGF-BB, FGF-2, and GDF-5 from vCMs coated with each of the respective recombinant growth factors was quantified in conditioned PBS solution collected from the matrices at the indicated time intervals over a 12-day period. Means ± SD from three independent experiments and significant differences, *** *p* < 0.001, ** *p* < 0.01, * *p* < 0.05, between experimental groups are shown. (**c**) A three-dimensional graph for the data present in (**b**) is used for better visualization of the peaks and speed (slope of the line) of growth factor release discussed in the text.

Combinations of biomaterials with bioactive substances have been reported as efficient treatment modalities in periodontal regenerative procedures [44,45]. Since protein adsorption to the biomaterial surface appears to be a key determinant of the behavior of cells grown on the biomaterial, we quantitatively investigated the amount of growth factors adsorbed to vCM as well as their release kinetics using an enzyme-linked immunosorbent assay (ELISA). We made the choice to coat the vCM with either enamel matrix derivative (EMD), which is widely used in periodontal regenerative surgery, or one of the following recombinant growth factors TGF-β1, PDGF-BB, FGF-2, or GDF-5 for a short, clinically relevant 10-min period. Our choice was partially based on the assumption that the majority of the listed proteins are released by activated platelets, and are thus naturally present in the blood clot [46,47]. Our results have shown that the vCM adsorbed each of the four growth factors with an efficiency higher than 93% (Figure 2a). The protein fraction that remained unadsorbed or was washed out, during the extensive rinsing procedure after the 10-min coating period, amounted to 3.4% on average across all four growth factors. Recombinant TGF-β1, PDGF-BB, and FGF-2 exhibited similar and significantly higher adsorption rates than the recombinant GDF-5. In addition, the adsorption of TGF-β1 was slightly but significantly higher compared to the adsorption of PDGF-BB and FGF-2.

The total protein release from TGF-β1- and PDGF-BB-coated matrices, expressed as percent of the adsorbed protein for the entire test period of 12 days, was 16.1 and 14.2%, respectively and appeared slightly but significantly higher than the total FGF-2 protein release amounting to 10.5% (Figure 2a). The release of GDF-5 occurred at a moderate but significantly higher rate of 29.4% compared to the other three factors.

Taken together, our data demonstrate excellent adsorption properties of the highly porous and crosslinked vCM. Furthermore, the total growth factor release for the entire test period was relatively low, in particular ≤ 30% of the adsorbed protein amounts, suggesting that enough adsorbed protein remained to be released over a period longer than 12 days.

### 2.4. Release Kinetics of Growth Factors from the Volume-Stable Collagen Matrix

Matrices coated with EMD were characterized by the release of TGF-β1 based on findings demonstrating TGF-β-like activity to be passively released from EMD-coated collagen membranes [48]. In general, the release of the investigated growth factors occurred in two phases. The peak of the growth factor release was arbitrary taken as the end of phase I whereas the period immediately after the peak release until day 12 was defined as phase II (Figure 2). GDF-5 and TGF-β1, the latter one released by TGF-β1-coated vCM, were characterized by a burst of release within 1 h for GDF-5 (1790 pg/mL) and 3 h for TGF-β1 (1224 pg/mL) (Figure 2b,c). On the contrary, PDGF-BB and FGF-2 as well as TGF-β1, released by EMD-coated vCM, were all characterized by a sustained release of smaller protein quantities within 6 h (710, 681, and 384 pg/mL for the three factors, respectively). Following the peak release at 3 h, TGF-β1, released by TGF-β1-coated vCM, was characterized by a quick drop in the release at 6 h, a second burst release at day 1, and a sustained release until the end of the test period. In contrast, following the gradually reached peak release, TGF-β1 released by EMD-coated vCM exhibited an equivalent gradual decrease up to day 3 and a second release peak at day 6.

Thus, two types of growth factor release kinetics were observed. In the first type, the highest amounts of the prominent promigratory and proangiogenic factors FGF-2 and PDGF-BB, equal to 64.1 and 56.3% of the total protein released within the 12-day period, respectively, were observed in the first 6 h spanning phase I (Figure 2a). In the second type, the highest amounts of GDF-5 and TGF-β1, equal to 60.8 and 58.3% of the total protein released within the 12-day period, respectively, were detected in the second release phase.

### 2.5. Increased Antifibrinolytic and Inhibited Profibrinolytic Gene Expression in Primary hPDLCs, hOFs, and HUVECs Grown on the Native/Uncoated Volume-Stable Collagen Matrix

**Figure 3:**
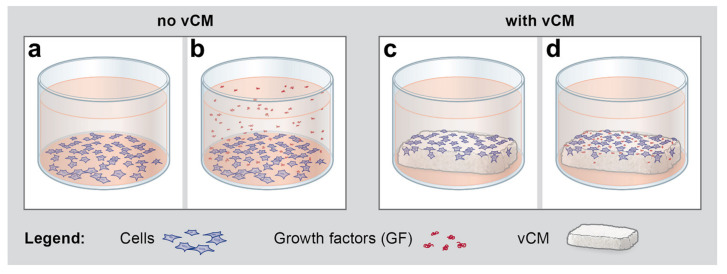
Schematic representation of the conditions under which hPDLCs, hOFs, and HUVECs were seeded for subsequent qRT-PCR analyses of the anti- and pro- fibrinolytic gene expression presented in Figure 4, Figure 5, and Figure 6. (**a**) Starved cells were grown for 48 h on tissue culture plastic in the absence of vCM and growth factors (GF), a condition further denoted as a control (Ctrl). (**b**) Starved cells were grown for 48 h on tissue culture plastic in the presence of GF applied in suspension. (**c**) Starved cells were grown for 48 h on native/uncoated vCM placed in ultra-low attachment plates. (**d**) Starved cells were grown for 48 h on vCM coated with EMD or each of the four recombinant growth factors, TGF-β1, PDGF-BB, FGF-2, or GDF-5 in ultra-low attachment plates. Please note that the scheme illustrates the conditions upon cell seeding and therefore, released from the vCM growth factors are not depicted in the cell culture medium in (**d**). In addition, there is no obvious difference in the appearance between tissue culture-treated plates for adhesive culture (**a,b**) and ultra-low attachment plates (**c,d**).

**Figure 4:**
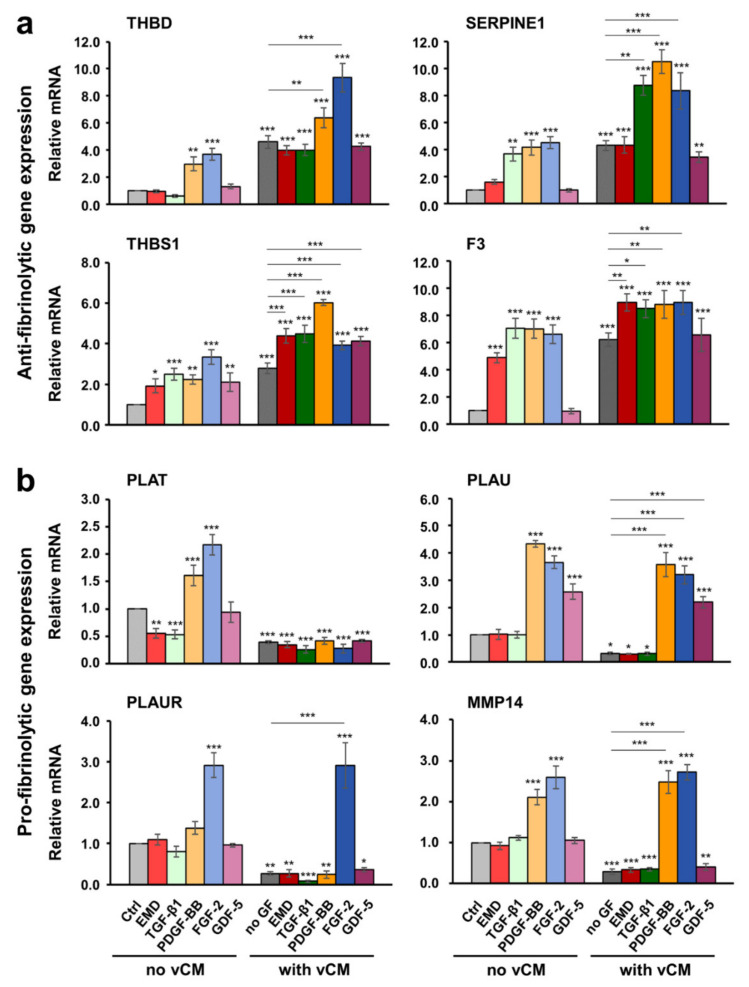
Effect of native/uncoated or growth factor-coated vCM on the anti- (**a**) and pro- fibrinolytic (**b**) gene expression in primary hPDLCs. Cells were grown under each of the four experimental conditions described in Figure 3 before total RNA was extracted and analyzed by qRT-PCR for the expression of THBD, SERPINE1, THBS1, and F3 antifibrinolytic (**a**) as well as PLAT, PLAU, PLAUR, and MMP14 profibrinolytic (**b**) genes. Values normalized to the housekeeping GAPDH are expressed relative to the values of control cells (Ctrl) grown as described in Figure 3a. Means ± SD from three independent experiments and significant differences to the Ctrl unless otherwise indicated, *** *p* < 0.001, ** *p* < 0.01, * *p* < 0.05 are shown.

As a next step, we investigated whether the native/uncoated or growth factor-coated vCM can modulate the fibrinolytic process. For this purpose, the expression of a number of anti- and pro- fibrinolytic genes was analyzed by qRT-PCR in cells cultured under four different conditions: (1) control condition, consisting of cells grown on tissue culture plastic in the absence of vCM and growth factors (Figure 3a), (2) cells grown on tissue culture plastic in the presence of EMD or growth factors applied in suspension (Figure 3b), (3) cells grown on native/uncoated vCM (Figure 3c), and (4) cells grown on vCM coated with EMD or each of the four recombinant growth factors, TGF-β1, PDGF-BB, FGF-2, or GDF-5 (Figure 3d).

**Figure 5:**
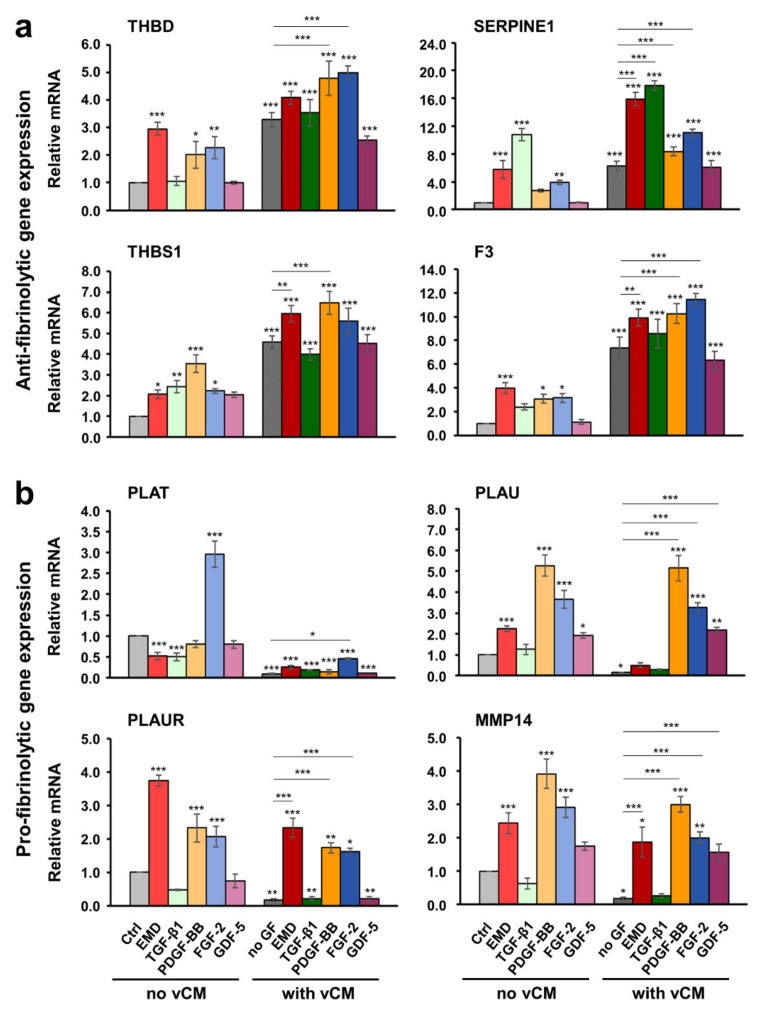
Effect of native/uncoated or growth factor-coated vCM on the anti- (**a**) and pro- fibrinolytic (**b**) gene expression in primary hOFs. Cells were grown under each of the four experimental conditions described in Figure 3 before total RNA was extracted and analyzed by qRT-PCR for the expression of THBD, SERPINE1, THBS1, and F3 antifibrinolytic (**a**) as well as PLAT, PLAU, PLAUR, and MMP14 profibrinolytic (**b**) genes. Values normalized to GAPDH are expressed relative to the values of control cells (Ctrl) grown as described in Figure 3a. Means ± SD from three independent experiments and significant differences to the Ctrl unless otherwise indicated, *** *p* < 0.001, ** *p* < 0.01, * *p* < 0.05 are shown.

**Figure 6:**
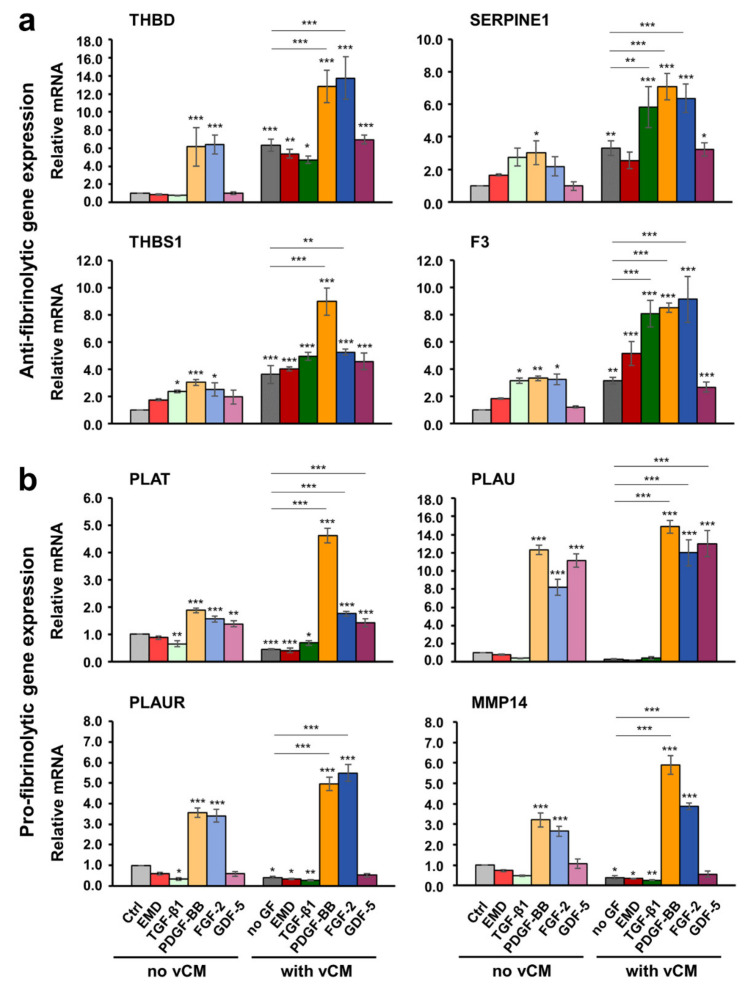
Effect of native/uncoated or growth factor-coated vCM on the anti- (**a**) and pro- fibrinolytic (**b**) gene expression in primary HUVECs. Cells were grown under each of the four experimental conditions described in Figure 3 before total RNA was extracted and analyzed by qRT-PCR for the expression of THBD, SERPINE1, THBS1, and F3 antifibrinolytic (**a**) as well as PLAT, PLAU, PLAUR, and MMP14 profibrinolytic (**b**) genes. Values normalized to GAPDH are expressed relative to the values of control cells (Ctrl) grown as described in Figure 3a. Means ± SD from three independent experiments and significant differences to the Ctrl unless otherwise indicated, *** *p* < 0.001, ** *p* < 0.01, * *p* < 0.05 are shown.

As a general trend, all three primary cell types, hPDLCs, hOFs and HUVECs grown on a native/uncoated vCM exhibited an extremely strong upregulation of the antifibrinolytic genes THBD, SERPINE1, THBS1, and F3 as well as a prominent downregulation of the profibrinolytic genes PLAT, PLAU, PLAUR, and MMP14, compared with control cells (Figure 4, Figure 5, and Figure 6; compare light and dark gray bars). The increase in the antifibrinolytic gene expression was statistically significant (*p* < 0.01), i.e., in the range of 3–7-fold for all three cell types (Figure 4a, Figure 5a, and Figure 6a). The decrease in the profibrinolytic gene expression was significant (*p* < 0.05), i.e., in the range of 6–10-fold in hOFs (Figure 5b) and 2–4-fold in hPDLCs (Figure 4b) and HUVECs (Figure 6b).

The observed changes in the gene expression indicate preserved biological activity of the growth factors released by the vCM.

### 2.6. Growth Factors Applied in Suspension or as a Coating on the Volume-Stable Collagen Matrix Exhibit Cell Type-Dependent Effects on Anti- and Pro-fibrinolytic Gene Expression. Potential Contribution of Growth factors to the Equilibrium between Coagulation and Fibrinolysis

In hPDLCs, PDGF-BB and FGF-2 applied in suspension prominently increased the expression of THBD, SERPINE1, THBS1, and F3 mRNA levels compared to control cells (Figure 4a). Furthermore, each of the two growth factors caused a statistically significant (*p* < 0.01) increase in the expression of all four antifibrinolytic genes in cells grown on the vCM compared to their expression in cells cultured on native/uncoated vCM. Moreover, a synergistic, greater than 2-fold increase in the expression of SERPINE1 and THBS1 above their already increased expression levels on native matrices was observed in hPDLCs cultured on PDGF-BB-coated matrices. A similar synergistic increase was detected for THBD and SERPINE1 in cells grown on FGF-2-coated vCM as well as for SERPINE1 in cells grown on TGF-β1-coated vCM. Among the four investigated antifibrinolytic genes, THBS1 was the only gene that appeared prominently induced by each of the examined bioactive substances including EMD, TGF-β1, and GDF-5, applied either in suspension or as a coating on vCM. Compared to the effect exhibited by the native/uncoated matrix, the effects of the matrices coated with each of the investigated active substances on the THBS1 mRNA levels were statistically better pronounced (*p* < 0.001). Along with THBS1, the expression levels of F3 were greatly induced by the EMD- or TGF-β1-coated vCMs by more than 8.5-fold compared with control cells. Interestingly, SERPINE1 remained the only gene, whose expression was upregulated in a synergistic manner by the combined activity of the matrix and a coating with either TGF-β1, PDGF-BB, or FGF-2.

Along with the strong statistically significant downregulation of PLAT, PLAU, PLAUR, and MMP14 in cells grown on native/uncoated vCM compared with control cells, we observed differential effects of some of the investigated growth factors when applied in suspension or as a coating to the matrix (Figure 4b). FGF-2 applied in suspension prominently increased the expression of all four profibrinolytic genes compared with their expression in control cells. However, whereas the vCM preserved the activity of the FGF-2 as an enhancer of the PLAU, PLAUR, and MMP14 mRNA expression in hPDLCs, the expression of PLAT remained strongly downregulated similar to its expression in hPDLCs grown on a native/uncoated matrix. The same was valid for the matrix-coated PDGF-BB, which significantly upregulated the expression of PLAU and MMP14 but not the expression of PLAT in hPDLCs grown on vCM. Along with PDGF-BB and FGF-2, GDF-5 was the only other growth factor that caused a significantly increased expression of PLAU when applied in suspension or as a coating to the vCM. Interestingly, among the four profibrinolytic genes, only PLAT was significantly (*p* < 0.01) downregulated by either EMD or recombinant TGF-β1 present in suspension. Moreover, the expression of all profibrinolytic genes remained significantly downregulated in cells cultured on EMD- or TGF-β1-coated matrices.

In general, in hOFs, the effects of the investigated growth factors on the expression of anti- and pro- fibrinolytic genes followed the trends seen in hPDLCs (Figure 5). For example, PDGF-BB and FGF-2, each of them applied either in suspension or as a coating to vCM, caused an increase in the expression of all four antifibrinolytic genes compared with their expression in control cells (Figure 5a). In contrast to hPDLCs and despite the fact that the SERPINE1 gene expression in hOFs appeared prominently induced by PDGF-BB and FGF-2 applied as a coating to vCM, the gene remained synergistically upregulated by the combined activity of the matrix and an EMD- or TGF-β1-coating only. As a general trend, a better pronounced effect of EMD applied in suspension or as a coating to vCM was observed in hOFs compared with the other two cell types. EMD exhibited a stimulatory effect on the expression of the majority of antifibrinolytic (Figure 5a) as well as profibrinolytic (Figure 5b) genes except for the expression of PLAT. On the analogy of hPDLCs, PLAT mRNA levels in hOFs were significantly downregulated (*p* < 0.001) by EMD- or TGF-β1 present in suspension (Figure 5b). Furthermore, they remained suppressed in hOFs cultured on EMD-, TGF-β1-, PDGF-BB-, or GDF-5-coated matrices. In contrast to hPDLCs, the strongly downregulated PLAT expression in hOFs cultured on uncoated matrices was reversed by FGF-2. Thus, the PLAT mRNA levels in cells grown on FGF-2-coated vCM were significantly (*p* < 0.05) upregulated by 4.7-fold compared with the PLAT expression in cells grown on an uncoated matrix. PDGF-BB, applied as a coating to the vCM, was also able to reverse the inhibitory effect of the uncoated vCM on the expression of PLAU, PLAUR, and MMP14 whereas the expression of PLAT remained significantly downregulated.

The anti- and pro- fibrinolytic gene expression in primary HUVECs was characterized with two trends: (1) with few exceptions, the stimulatory effect of the vCM on the expression of the antifibrinolytic genes was synergistically enhanced by TGF-β1, PDGF-BB, or FGF-2; and (2) the strong inhibitory effect of the vCM on the expression of profibrinolytic genes was completely reversed by PDGF-BB, FGF-2, or GDF-5 (except for PLAUR and MMP14) (Figure 6). In particular, PDGF-BB, applied as a coating on the matrix, significantly (*p* < 0.001) upregulated the expression of all four antifibrinolytic genes by 2.0–2.7-fold above the increased expression observed in cells grown on a native/uncoated matrix (Figure 6a). FGF-2 coated on the vCM was able to cause a strong (*p* < 0.001) upregulation of THBD and F3 mRNA levels by 2.2- and 2.9-fold, respectively, above the expression levels detected on native/uncoated matrices. Finally, the F3 gene expression, which was significantly (*p* < 0.001) increased by 3.1-fold in HUVECs grown on uncoated vCMs, reached an 8.1-fold increase in cells grown on TGF-β1-coated matrices. In contrast to the two oral cell types, no effect of EMD on the gene expression was detected in HUVECs grown in the absence or presence of a matrix (Figure 6a,b).

In terms of profibrinolytic gene expression, PDGF-BB and FGF-2 applied in suspension had an extremely strong (*p* < 0.001) stimulatory effect on the expression of all four profibrinolytic genes whereas GDF-5 applied in suspension was able to significantly (*p* < 0.01) upregulate the expression of PLAT and PLAU only (Figure 6b). The application of the growth factors as a coating on the vCM was able to completely reverse the strong inhibitory effect of the native matrices on the expression of the profibrinolytic genes. In particular, cells grown on PDGF-BB- or FGF-2-coated matrices were characterized with an extremely significant (*p* < 0.001) increase in the expression levels of PLAT, PLAU, PLAUR, and MMP14 genes. The increase was in folds higher than the inhibited expression of the genes in cells cultured on native matrices. The same effect was exhibited by the GDF-5 on the expression of PLAT and PLAU.

Taken together, our data illustrate that for the most part, the antifibrinolytic and thus, clot stabilizing effects of the investigated vCM were synergistically enhanced by growth factors, especially in primary HUVECs. The clot stabilization was further supported by a strong suppression of the profibrinolytic gene expression observed on native matrices. However, with small exceptions, growth factors applied as a coating on the matrices were able to reverse this inhibitory effect, thereby potentially contributing to the equilibrium between coagulation and fibrinolysis.

## 3. Discussion

**Figure 7:**
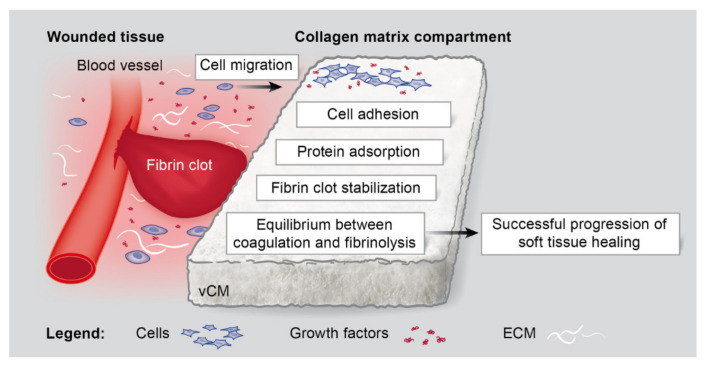
Schematic representation of the effects exhibited by vCM on the early events of the wound healing process. The ability of the vCM to affect the cellular migration and adhesion, protein adsorption, fibrin clot stabilization, and balance between coagulation and fibrinolysis, all as prerequisites for the successful continuation of the soft tissue healing process, are depicted.

Various collagen-based soft tissue substitute materials have been introduced in periodontal and peri-implant soft tissue reconstructive surgery. A number of clinical studies comparing autogenous CTGs with collagen-based matrices have noted the superiority of the autografts, especially in achieving an optimal thickness of the regenerated tissue [2,5,15,16,17,18,19,49]. The present study focused on a highly porous, volume-stable collagen matrix [21,23,24,25,26,27,28], which is able to overcome the volume-stability limitation of most commercially available grafts. During reconstructive surgery, the soft tissue substitute material comes into a contact with blood, leading to clot formation on its surface as the very first step of the wound healing process. A blood clot, constituted by activated platelets, neutrophils, and erythrocytes, embedded in a matrix of fibrin, serves as a scaffold for the adhesion of oral fibroblasts, endothelial cells, leucocytes, and macrophages. The fibrin clot also serves as a natural reservoir for growth factors [30], which the collagen-based matrices are able to efficiently bind [7]. This means that the efficiency with which the fibrin clot is formed as well as its stability and longevity will be determining factors for the pre-activation/biofunctionalization of the grafting material. Subsequent dissolution of the fibrin clot (fibrinolysis), once it has served its function in hemostasis, will ensure the progression to the next stages of the wound healing process. Despite the key role of the fibrin clot in supporting the soft tissue regeneration, assessments of the capacity of biomaterials to stabilize the fibrin clot are largely missing. A scheme that summarizes our findings for the effects of the investigated vCM on the early events of the wound healing process is presented in Figure 7. Our data have convincingly demonstrated enhanced promigratory and proadhesive properties of three primary cell types (hPDLCs, hOFs, and HUVECs), grown on the vCM. The vCM was characterized with an efficient adsorption of four recombinant growth factors (TGF-β, PDGF-BB, FGF-2, and GDF-5), naturally present in the blood clot, after a clinically relevant, 10-min incubation period. Notably, a sustained release of growth factors with preserved biological activity was evident. Finally, the ability of the vCM to stabilize the fibrin clot was manifested by upregulation of antifibrinolytic and downregulation of profibrinolytic gene expression in cells grown on the matrix. Moreover, cells grown on the vCM attempted to maintain an equilibrium between coagulation and fibrinolysis as parts of the hemostatic process. An optimal proteolytic balance achieved by the concomitant expression of proteases and protease inhibitors is important for degradation of the extracellular matrix that in its turn is required for successful angiogenesis, re-epithelialization, and tissue remodeling to occur.

Growth factor release from the vCM occurred in two phases. In the first phase, the highest release from the matrix was observed at an early time point, whereas the second phase, spanning the period immediately after the peak of the release until day 12, was likely corresponding to the delayed release of the growth factors from the deeper vCM layers. Interestingly, two types of release kinetics were evident. The proangiogenic factors FGF-2 and PDGF-BB were characterized with a higher release in phase I spanning the first 6 h, whereas GDF-5 and TGF-β1 dominated in the second phase. Specific binding of the growth factors to the reconstituted collagens in the vCM may underlie the specific time pattern of growth factor release and bioavailability, respectively. The first type of release kinetics is more likely to serve the initial early phases of the soft tissue healing, which require fast recruitment of various cell types to the wounded site, whereas the second type of release kinetics may appear advantageous for the longer tissue remodeling/maturation phase of the regenerative process. This assumption is supported by a number of studies demonstrating that binding of growth factors to extracellular matrix components, including collagens, may localize and modulate their biological activities [50].

Some of the anti- and pro- fibrinolytic factors investigated in the current study are also known to influence the migratory, proliferative, and differentiation potential of cells. For instance, a gene set enrichment analysis of lung tissues from recombinant thrombomodulin-treated mice in a vascular endothelial injury model showed an upregulated expression of genes involved in cell proliferation, cell differentiation, and anti-inflammatory processes [51]. Investigation of these processes in relation to vCM deserves special attention. It may be speculated that the increased expression of THBD encoding thrombomodulin in cells cultured on native/uncoated as well as growth factor-coated vCMs will reduce the inflammation at the wounded site and facilitate progression to the next proliferative stage of the healing process. Indeed, accelerated healing of radiation burn lesions by gingival fibroblasts, injected intradermally in mice, was partially attributed to strong overexpression of THBD compared to its expression in transplanted mesenchymal stem cells [52]. The synergism between the effects of vCM and PDGF-BB on the THBD expression was especially pronounced in HUVECs whereas the synergism between the vCM and FGF-2 in hPDLCs. Administration of recombinant FGF-2 has been shown to increase the expression of thrombomodulin in HUVEC medium [53]. In vascular smooth muscle cells, PDGF-BB-induced expression of thrombomodulin enhanced cell migration and spreading onto type I collagen [54], which is a predominant collagen type in the vCM too. Furthermore, it has been suggested that the PDGF-BB-regulated thrombomodulin expression may play a prominent role in vascular remodeling [55]. Thus, investigation of the effect of PDGF-BB- or FGF-2-coated vCM on the angiogenic and vascular remodeling potential of endothelial cells is warranted.

The plasminogen activator inhibitor type 1 (PAI-1), encoded by the SERPINE1 gene, is a major physiological inhibitor of fibrinolysis and proteolytic processes associated with wound healing [56]. Interestingly, it has been shown that recombinant human PAI-1-treated PDLCs transplanted subcutaneously in a mouse model were able to form cementum-like tissue with insertion of periodontal ligament fibers [57]. This suggests that recombinant human PAI-1 may be a good candidate for clinical applications in periodontal tissue regeneration. In this respect, our results of strongly induced SERPINE1 gene expression in primary hPDLCs, hOFs, and HUVECs grown on native as well as TGF-β1-, PDGF-BB- or FGF-2-coated vCMs strongly support the favorable effect of vCM on the regenerative process. The three growth factors TGF-β1, PDGF-BB, and FGF-2 as well as the investigated by us thrombospondin 1, encoded by the THBS1 gene in humans, are released by activated platelets at wounded sites [46,47,58]. All four factors are known to efficiently bind extracellular matrix proteins such as collagen [7,59] and thus, they may coat the vCM in situ after its placement at the defect site. In agreement with our data demonstrating an upregulated expression of THBS1 mRNA in response to TGF-β1, PDGF-BB, or FGF-2 applied to the primary oral fibroblasts or endothelial cells in suspension, others have also shown a stimulatory effect of the three growth factors on THBS1 expression in various cell types [60,61]. However, the effects of thrombospondin 1 in relation to the growth factors are often reciprocal. Thrombospondin 1 binds each of the three growth factors with high affinity and in its turn affects their bioavailability and activity. Numerous studies have shown that thrombospondin 1 activates latent TGF-β1 in various cell types, including endothelial cells, fibroblasts, and macrophages [62]. Binding of thrombospondin 1 to PDGF protected the latter from proteolytic degradation, hence increasing its stability, bioavailability, migratory, and proliferative effects on mesenchymal stromal cells [63]. Furthermore, thrombospondin 1 stimulated vascular remodeling by increasing PDGF-BB as well as its own expression [64]. In this respect, the synergistic effect of the vCM and PDGF-BB on the expression of THBS1 that we observed in all three cell lines, might partially be attributed to the reciprocal activity of thrombospondin 1.

The thromboplastin or tissue factor, encoded by the F3 gene in humans, is a transmembrane glycoprotein with a key role in coagulation. The intracellular part of the tissue factor contains two putative phosphorylation sites, which turns it into a mediator of intracellular signaling events altering gene expression patterns and cellular behavior [35]. The observed by us strong synergism in the effects of the vCM and each of the growth factors TGFβ1, PDGF-BB, or FGF-2 on the F3 gene expression in HUVECs suggests a potential alteration in the behavior of the endothelial cells grown on the matrix that deserves further investigation.

Like the coagulation, a number of proteins tightly regulate fibrinolysis [31]. Plasmin is the key fibrinolysin, which is activated from plasminogen by one of two proteases, i.e., tissue plasminogen activator (tPA) encoded by the human PLAT gene and urokinase-type plasminogen activator (uPA) encoded by the human PLAU gene. The activity of uPA is restricted to the cell surface by the urokinase receptor encoded by the human PLAUR gene. In the absence of plasminogen, MMP14, also known as MT1-MMP, also acts as a fibrinolysin [37]. The antifibrinolytic activity of the vCM reported in the present study was supported by strong downregulation of the genes encoding the above listed profibrinolytic factors in cells grown on native matrices. Notably, most of the investigated growth factors applied as a coating on the vCM were able to completely reverse this inhibition. An exception was seen in the expression of PLAT that remained strongly inhibited by the vCMs coated with each of the growth factors in hPDLCs but was considerably reversed by the FGF-2-coated vCM in hOFs as well as by the PDGF-BB-, FGF-2-, and GDF-5-coated vCMs in HUVECs. This suggests a cell type-specific regulation of PLAT expression by the various growth factors coated on the vCM, which deserves further investigation. Others have also observed that exposure of periodontal ligament cells and gingival fibroblasts to PDGF-BB resulted in increased PLAU and SERPINE1 transcripts but not of PLAT [65]. In conclusion, vCM that has naturally adsorbed growth factors contained in the surrounding wounded tissue or growth factors made de novo by cells attracted in the matrix compartment will certainly contribute to the balance between coagulation and fibrinolysis by enhancing the expression of both anti- and pro- fibrinolytic genes (Figure 7).

A major limitation of the present study is the use of traditional cell monolayers as control and the lack of comparative nature. The utilization of commercially available gelatinous protein mixtures, such as Matrigel, were not considered due to their specific protein composition, characteristic of basement membranes, and the presence of bioactive substances even in reduced growth factor formulations. Collagen hydrogels, as another class of three-dimensional materials, have high water content and low mechanical strength compared to the porous vCM. The properties of the hydrogels are highly variable and depend on a large number of parameters such as collagen source, solubilization method, polymerization pH, polymerization temperature, ionic strength, and collagen concentration [66]. This variability limits their use as a 3D scaffold-based control. Furthermore, no other volume-stable biomaterials indicated for soft tissue augmentation in periodontal reconstruction surgery were included in the study. This limitation can be addressed in future in vitro, preclinical, and clinical studies.

The combination between enhanced migratory and adhesive properties of cells grown on the vCM and the valuable equilibrium between coagulation and fibrinolysis, guaranteed by an optimal growth factor adsorption and release profile of the vCM, ensures successful progression to the next stages of the soft tissue healing process in the presence of vCM.

## 4. Materials and Methods

### 4.1. Cell Culture

Primary hPDLCs and hOFs were obtained from three donors each using the tissue explant technique as described previously [9]. The collection of donor tissue from systemically and periodontally healthy individuals below 35 years had received an approval by the Ethics Committee, Berne, Switzerland (ethical code ID 2018-00661 from 13.08.2018). Primary cells derived from 1mm-sized tissue explant pieces were cultured in Dulbecco’s Modified Eagle Medium (DMEM; ThermoFisher Scientific, Basel, Switzerland) supplemented with 10% fetal calf serum (FCS; ThermoFisher Scientific) and 1% antimycotics/antibiotics (AA; ThermoFisher Scientific). Primary HUVECs [PCS100013; American Type Culture Collection (ATCC), Manassas, VA, USA], pooled from ten individual donors, were propagated in medium 199 (M199; ThermoFisher Scientific) supplemented with 10% FCS, 12 μg/mL bovine brain extract (Lonza, Visp, Switzerland), 10 ng/mL human recombinant endothelial growth factor (EGF; Peprotech, London, UK), 25 U/mL heparin (Sigma, Buchs, Switzerland), 1 μg/mL hydrocortisone (Sigma), 2 mM l-glutamine (ThermoFisher Scientific) and 1% AA. Primary cells that had not undergone more than seven passages were starved in 0.3% FCS-containing medium before culturing under experimental conditions.

Geistlich Fibro-Gide^®^ matrices (vCM) in the 15 × 20 × 6 mm range size were kindly provided by Geistlich Pharma (Wolhusen, Switzerland). Matrices were cut sterile into 7.5 × 10 mm pieces, shortly rinsed in cell culture medium, and placed on the bottom of ultra-low attachment 24-well plates (Corning, NY, USA). In some cases, EMD (Straumann^®^ Emdogain^®^; botiss biomaterials GmbH, Zossen, Germany) or each of the recombinant growth factors TGF-β1, PDGF-BB, FGF-2, or GDF-5 (Peprotech) were used in suspension or applied as a coating to the vCM. Cells seeded directly on the tissue culture-treated surface of 24-well plates for adherent cell culture (Greiner Bio-One, St. Gallen, Switzerland), in the absence of EMD or growth factors, were used as control (Ctrl).

### 4.2. Cell Migration Assay

Cell migration was analyzed by a modified Boyden chamber migration assay using transwell permeable supports (6.5 mm diameter; 8 µm pore size; Greiner Bio-One) as described previously [67]. After 24 h of starvation, 5 × 10^4^ cells were seeded in the top insert chamber with 200 µL serum-free M199 or DMEM. The vCM with a size of 7.5 × 10 × 3 mm was placed in the low chamber with 800 µL 10% FCS-containing M199 or DMEM. Cells were allowed to migrate across the permeable support for 17 h at 37°C before fixation in 10% neutral buffered formalin (Shandon Formal-Fixx Concentrate; ThermoFisher Scientific), and staining in 0.1% crystal violet solution (Sigma). Image acquisition was performed on an Olympus CKX41 microscope. Migration was quantified by using the ImageJ software as described previously [67]. Data represent means ± SD from three independent experiments performed in duplicates with the pooled HUVECs or with three different cell donors for the primary hPDLCs and hOFs.

### 4.3. Cell Adhesion Assay

The adhesion properties of primary hPDLCs, hOFs, and HUVECs plated on vCM were determined by crystal violet staining of adherent cells as described previously [68]. In brief, 6.25 × 10^5^ cells/well were seeded on tissue culture-treated surface (control) or vCM placed in ultra-low attachment 24-well plates. The cells were allowed to adhere for 1, 3, and 6 h before removal of nonadherent cells by 6 washing cycles in PBS, followed by fixation in 10% neutral buffered formalin (Shandon Formal-Fixx Concentrate) and staining in 0.1% crystal violet solution. The two last steps were performed for 30 min each at room temperature (RT). After 8 washing cycles in deionized H_2_O and prompt 20 s immersion of the matrices in 10% (*v*/*v*) acetic acid (Sigma) to remove nonspecific crystal violet staining, the dye bound to adherent cells was solubilized in the latter solution on an orbital shaker at 150 rpm for 5 min at RT. To correct for a potential background staining in both control and vCM experimental groups, no-cell samples were included in the assay. To obtain a value for the total number of plated cells, cells were allowed to settle down for 1 h and then directly fixed (omitting the six washing steps) in 10% neutral buffered formalin. The rest of the procedure was followed as described above. The absorbance of the eluate was measured at 450 nm using an EL808 reader (BioTek Instruments GmbH, Lucerne, Switzerland). Experimental values corrected for the background signal were normalized to the values obtained for the total number of seeded control cells, arbitrary taken as 100% adhesion. Data represent means ± SD from three independent experiments performed with the pooled HUVECs or with three different cell donors for the primary hPDLCs and hOFs.

### 4.4. Adsorption and Release of Growth Factors from Volume-Stable Collagen Matrix

The vCMs were coated for 10 min at RT with 1 mg/mL of EMD or 100 ng/mL of each of the following recombinant growth factors TGF-β1, PDGF-BB, FGF-2, or GDF-5. The EMD and the growth factors were diluted in PBS, pH 7.2 from a 10 mg/mL and 10 µg/mL stock solutions, respectively. vCM incubated in PBS alone was used as a control. To remove unbound protein after the 10-min incubation, the vCMs were extensively washed with PBS for three cycles of 5 min each. The washing solutions from all three cycles for each of the growth factors were pooled and stored in low protein binding tubes (Eppendorf, Basel, Switzerland) at −80 °C until analyzed. Matrices were transferred in 3 mL PBS containing 0.1% bovine serum albumin (BSA; Sigma) and 1% AA for incubation at 37 °C for 12 days with shaking at 70 rpm. The 3-mL supernatants were collected in low protein binding tubes, stored at −80 °C, and replaced with 3 mL of fresh PBS/BSA/AA solution at 0.5, 1, 3, and 6 h, and 1, 3, 6, 9, and 12 days. The amount of protein remained unadsorbed to the vCM as well as the protein released at the indicated time points was determined by colorimetric ELISAs (R&D Systems, Minneapolis, MN, USA) following the manufacturer’s protocol. The optical density was read at 450 nm wave length using the EL808 microplate reader. Growth factor adsorption by the vCM was quantified by subtracting the amount of unbound growth factor (determined in the washing solution) from the total amount of protein initially added to the matrices (100 ng), and was expressed in percent. The amount of released protein was calculated as percent of the adsorbed protein. Three independent experiments were performed. Samples were quantified in triplicate.

### 4.5. Gene Expression Analyses by qRT-PCR

Quantitative RT-PCR was used to analyze the expression of THBD, SERPINE1, THBS1, F3, PLAT, PLAU, PLAUR, and MMP14 genes in primary hPDLCs, hOFs, and HUVECs grown for 48 h under the experimental conditions depicted in Figure 3. In brief, after 24 h of starvation, 2.5 × 10^5^ cells/well were plated in 1% FCS-containing medium either (1) on tissue culture plastic in the absence of vCM and growth factors (control), (2) on tissue culture plastic in the presence of 1 mg/mL of EMD or 100 ng/mL of TGF-β1, PDGF-BB, FGF-2, or GDF-5, (3) on native/uncoated vCM, or (4) on vCM coated with EMD or each of the four recombinant growth factors, according to the coating procedure described in Section 4.4.

Total cellular RNA from the above-described experimental groups was extracted using TRIzol (ThermoFisher Scientific) according to the manufacturer’s procedure. The TRIzol-extracted RNA was purified by using the RNeasy MinElute Cleanup Kit (Qiagen, Basel, Switzerland). RNA, quantified spectrophotometrically and reverse transcribed by using the Transcriptor First Strand cDNA Synthesis Kit (Roche, Basel, Switzerland), was subjected to qPCR using the FastStart Universal SYBR Green Master ROX (Roche) and the primer sequences listed in Table 1. The PCR was carried out in a QuantStudio 3 instrument (Applied Biosystems, Rotkreuz, Switzerland) as described [9]. The efficiency ∆∆Ct method was used to calculate gene expression levels normalized to GAPDH and calibrated to controls. Data represent means ± SD from three independent experiments performed in duplicates with the pooled HUVECs or with three different cell donors for the primary hPDLCs and hOFs.

### 4.6. Statistical Analysis

Means ± SD represent all grouped data. The statistical analyses were carried out using GraphPad InStat Software, v.3.05. Differences between two groups were assessed by Student’s *t* test and between multiple groups by one-way analysis of variance (ANOVA) with Tukey’s post hoc test. Significance was indicated using the scale, *** *p* < 0.001, ** *p* < 0.01 and * *p* < 0.05.

## Figures and Tables

**Table 1:** Primer sequences.

Gene Symbol	Gene Bank Accession Number	Primer Pair (fwd/rev)	Amplicon Size (bp)
THBD	NM_000361	5′- TCCTCTGCGAGTTCCACTTC-3’ 5′- GCCGTAGGTGATCGAGACG-3’	89
SERPINE1	NM_001165413	5′- AGTGGACTTTTCAGAGGTGGA-3’ 5′- GCCGTTGAAGTAGAGGGCATT-3’	151
THBS1	NM_003246.3	5′- ATCCGCAAAGTGACTGAAGAG-3’ 5′- GCAGATGGTAACTGAGTTCTGAC-3’	152
F3	NM_001993	5′- TAAGCACTAAGTCAGGAGATTGG-3’ 5′- TGTCCGAGGTTTGTCTCCAG-3’	216
PLAT	NM_000930	5′- AAACCCAGATCGAGACTCAAAGC-3’ 5′- GGTAGGCTGACCCATTCCC-3’	131
PLAU	NM_001145031	5′- GGGAATGGTCACTTTTACCGAG-3’ 5′- GGGCATGGTACGTTTGCTG-3’	103
PLAUR	NM_001005377	5′- GAGCTATCGGACTGGCTTGAA-3’ 5′- CGGCTTCGGGAATAGGTGAC-3’	108
MMP14	NM_004995.3	5′- TCAAAGGAGACAAGCATTGGG-3’ 5′- CGGTAGTACTTGTTTCCACGG-3’	165
GAPDH	NM_001256799.2	5′- ATCAAGAAGGTGGTGAAGCAG-3’ 5′- TCGTTGTCATACCAGGAAATGAG-3’	178

## Data Availability

All data generated and analyzed during this study are included in this article.

## References

[1] Vignoletti F., Nunez J., Sanz M. (2014). Soft tissue wound healing at teeth, dental implants and the edentulous ridge when using barrier membranes, growth and differentiation factors and soft tissue substitutes. J. Clin. Periodontol..

[2] Tonetti M.S., Cortellini P., Pellegrini G., Nieri M., Bonaccini D., Allegri M., Bouchard P., Cairo F., Conforti G., Fourmousis I. (2018). Xenogenic collagen matrix or autologous connective tissue graft as adjunct to coronally advanced flaps for coverage of multiple adjacent gingival recession: Randomized trial assessing non-inferiority in root coverage and superiority in oral health-related quality of life. J. Clin. Periodontol..

[3] Cosgarea R., Juncar R., Arweiler N., Lascu L., Sculean A. (2016). Clinical evaluation of a porcine acellular dermal matrix for the treatment of multiple adjacent class I, II, and III gingival recessions using the modified coronally advanced tunnel technique. Quintessence Int..

[4] Shirakata Y., Sculean A., Shinohara Y., Sena K., Takeuchi N., Bosshardt D.D., Noguchi K. (2016). Healing of localized gingival recessions treated with a coronally advanced flap alone or combined with an enamel matrix derivative and a porcine acellular dermal matrix: A preclinical study. Clin. Oral Investig..

[5] Pietruska M., Skurska A., Podlewski Ł., Milewski R., Pietruski J. (2019). Clinical evaluation of Miller class I and II recessions treatment with the use of modified coronally advanced tunnel technique with either collagen matrix or subepithelial connective tissue graft: A randomized clinical study. J. Clin. Periodontol..

[6] Tavelli L., McGuire M.K., Zucchelli G., Rasperini G., Feinberg S.E., Wang H.-L., Giannobile W.V. (2020). Extracellular matrix-based scaffolding technologies for periodontal and peri-implant soft tissue regeneration. J. Periodontol..

[7] Wang A.Y., Leong S., Liang Y.-C., Huang R.C.C., Chen C.S., Yu S.M. (2008). Immobilization of growth factors on collagen scaffolds mediated by polyanionic collagen mimetic peptides and its effect on endothelial cell morphogenesis. Biomacromolecules.

[8] Nica C., Lin Z., Sculean A., Asparuhova M.B. (2020). Adsorption and release of growth factors from four different porcine-derived collagen matrices. Materials.

[9] Lin Z., Nica C., Sculean A., Asparuhova M.B. (2020). Enhanced Wound Healing Potential of Primary Human Oral Fibroblasts and Periodontal Ligament Cells Cultured on Four Different Porcine-Derived Collagen Matrices. Materials.

[10] Fischer K.R., Fickl S., Mardas N., Bozec L., Donos N. (2014). Stage-two surgery using collagen soft tissue grafts: Clinical cases and ultrastructural analysis. Quintessence Int..

[11] Sculean A., Mihatovic I., Shirakata Y., Bosshardt D.D., Schwarz F., Iglhaut G. (2015). Healing of localized gingival recessions treated with coronally advanced flap alone or combined with either a resorbable collagen matrix or subepithelial connective tissue graft. A preclinical study. Clin. Oral Investig..

[12] Moreira A.R.O., Santamaria M.P., Silvério K.G., Casati M.Z., Nociti Junior F.H., Sculean A., Sallum E.A. (2016). Coronally advanced flap with or without porcine collagen matrix for root coverage: A randomized clinical trial. Clin. Oral Investig..

[13] Schmitt C.M., Moest T., Lutz R., Wehrhan F., Neukam F.W., Schlegel K.A. (2016). Long-term outcomes after vestibuloplasty with a porcine collagen matrix (Mucograft(^®^)) versus the free gingival graft: A comparative prospective clinical trial. Clin. Oral Implants Res..

[14] Rotundo R., Genzano L., Patel D., D’Aiuto F., Nieri M. (2019). Adjunctive benefit of a xenogenic collagen matrix associated with coronally advanced flap for the treatment of multiple gingival recessions: A superiority, assessor-blind, randomized clinical trial. J. Clin. Periodontol..

[15] De Resende D.R.B., Greghi S.L.A., Siqueira A.F., Benfatti C.A.M., Damante C.A., Ragghianti Zangrando M.S. (2019). Acellular dermal matrix allograft versus free gingival graft: A histological evaluation and split-mouth randomized clinical trial. Clin. Oral Investig..

[16] Cieślik-Wegemund M., Wierucka-Młynarczyk B., Tanasiewicz M., Gilowski Ł. (2016). Tunnel Technique With Collagen Matrix Compared With Connective Tissue Graft for Treatment of Periodontal Recession: A Randomized Clinical Trial. J. Periodontol..

[17] Tavelli L., Barootchi S., Di Gianfilippo R., Modarressi M., Cairo F., Rasperini G., Wang H.L. (2019). Acellular dermal matrix and coronally advanced flap or tunnel technique in the treatment of multiple adjacent gingival recessions. A 12-year follow-up from a randomized clinical trial. J. Clin. Periodontol..

[18] Matoh U., Petelin M., Gašperšič R. (2019). Split-Mouth Comparison of Coronally Advanced Flap with Connective Tissue Graft or Collagen Matrix for Treatment of Isolated Gingival Recessions. Int. J. Periodontics Restor. Dent..

[19] Wei P.C., Laurell L., Lingen M.W., Geivelis M. (2002). Acellular dermal matrix allografts to achieve increased attached gingiva. Part 2. A histological comparative study. J. Periodontol..

[20] Núñez J., Caffesse R., Vignoletti F., Guerra F., San Roman F., Sanz M. (2009). Clinical and histological evaluation of an acellular dermal matrix allograft in combination with the coronally advanced flap in the treatment of Miller class I recession defects: An experimental study in the mini-pig. J. Clin. Periodontol..

[21] Thoma D.S., Zeltner M., Hilbe M., Hämmerle C.H., Hüsler J., Jung R.E. (2016). Randomized controlled clinical study evaluating effectiveness and safety of a volume-stable collagen matrix compared to autogenous connective tissue grafts for soft tissue augmentation at implant sites. J. Clin. Periodontol..

[22] Mathes S.H., Wohlwend L., Uebersax L., von Mentlen R., Thoma D.S., Jung R.E., Görlach C., Graf-Hausner U. (2010). A bioreactor test system to mimic the biological and mechanical environment of oral soft tissues and to evaluate substitutes for connective tissue grafts. Biotechnol. Bioeng..

[23] Thoma D.S., Jung R.E., Schneider D., Cochran D.L., Ender A., Jones A.A., Görlach C., Uebersax L., Graf-Hausner U., Hämmerle C.H. (2010). Soft tissue volume augmentation by the use of collagen-based matrices: A volumetric analysis. J. Clin. Periodontol..

[24] Ferrantino L., Bosshardt D., Nevins M., Santoro G., Simion M., Kim D. (2016). Tissue Integration of a Volume-Stable Collagen Matrix in an Experimental Soft Tissue Augmentation Model. Int. J. Periodontics Restorative Dent..

[25] Zeltner M., Jung R.E., Hämmerle C.H., Hüsler J., Thoma D.S. (2017). Randomized controlled clinical study comparing a volume-stable collagen matrix to autogenous connective tissue grafts for soft tissue augmentation at implant sites: Linear volumetric soft tissue changes up to 3 months. J. Clin. Periodontol..

[26] Caballé-Serrano J., Zhang S., Ferrantino L., Simion M., Chappuis V., Bosshardt D.D. (2019). Tissue Response to a Porous Collagen Matrix Used for Soft Tissue Augmentation. Materials.

[27] Caballé-Serrano J., Zhang S., Sculean A., Staehli A., Bosshardt D.D. (2020). Tissue Integration and Degradation of a Porous Collagen-Based Scaffold Used for Soft Tissue Augmentation. Materials.

[28] Imber J.C., Bosshardt D.D., Stähli A., Saulacic N., Deschner J., Sculean A. (2021). Pre-clinical evaluation of the effect of a volume-stable collagen matrix on periodontal regeneration in two-wall intrabony defects. J. Clin. Periodontol..

[29] Sculean A., Gruber R., Bosshardt D.D. (2014). Soft tissue wound healing around teeth and dental implants. J. Clin. Periodontol..

[30] Tezono K., Sarker K.P., Kikuchi H., Nasu M., Kitajima I., Maruyama I. (2001). Bioactivity of the vascular endothelial growth factor trapped in fibrin clots: Production of IL-6 and IL-8 in monocytes by fibrin clots. Haemostasis.

[31] Chapin J.C., Hajjar K.A. (2015). Fibrinolysis and the control of blood coagulation. Blood Rev..

[32] Mosher D.F., Misenheimer T.M., Stenflo J., Hogg P.J. (1992). Modulation of fibrinolysis by thrombospondin. Ann. N. Y. Acad. Sci..

[33] Anonick P.K., Yoo J.K., Webb D.J., Gonias S.L. (1993). Characterization of the antiplasmin activity of human thrombospondin-1 in solution. Biochem. J..

[34] Poon I.K., Patel K.K., Davis D.S., Parish C.R., Hulett M.D. (2011). Histidine-rich glycoprotein: The Swiss Army knife of mammalian plasma. Blood.

[35] Witkowski M., Landmesser U., Rauch U. (2016). Tissue factor as a link between inflammation and coagulation. Trends Cardiovasc. Med..

[36] Urano T., Suzuki Y., Iwaki T., Sano H., Honkura N., Castellino F.J. (2019). Recognition of Plasminogen Activator Inhibitor Type 1 as the Primary Regulator of Fibrinolysis. Curr. Drug Targets.

[37] Cesarman-Maus G., Hajjar K.A. (2005). Molecular mechanisms of fibrinolysis. Br. J. Haematol..

[38] Pluskota E., Soloviev D.A., Plow E.F. (2003). Convergence of the adhesive and fibrinolytic systems: Recognition of urokinase by integrin alpha Mbeta 2 as well as by the urokinase receptor regulates cell adhesion and migration. Blood.

[39] Franchini M., Mannucci P.M. (2018). Primary hyperfibrinolysis: Facts and fancies. Thromb. Res..

[40] Juhan-Vague I., Alessi M.C., Morange P.E. (2000). Hypofibrinolysis and increased PAI-1 are linked to atherothrombosis via insulin resistance and obesity. Ann. Med..

[41] Mah W., Jiang G., Olver D., Cheung G., Kim B., Larjava H., Häkkinen L. (2014). Human Gingival Fibroblasts Display a Non-Fibrotic Phenotype Distinct from Skin Fibroblasts in Three-Dimensional Cultures. PLoS ONE.

[42] Zhu W., Liang M. (2015). Periodontal ligament stem cells: Current status, concerns, and future prospects. Stem Cells Int..

[43] Bachetti T., Morbidelli L. (2000). Endothelial cells in culture: A model for studying vascular functions. Pharmacol. Res..

[44] Cochran D.L., Oh T.J., Mills M.P., Clem D.S., McClain P.K., Schallhorn R.A., McGuire M.K., Scheyer E.T., Giannobile W.V., Reddy M.S. (2016). A randomized clinical trial evaluating rh-FGF-2/β-TCP in periodontal defects. J. Dent. Res..

[45] Sangiorgio J.P.M., Neves F., Rocha Dos Santos M., França-Grohmann I.L., Casarin R.C.V., Casati M.Z., Santamaria M.P., Sallum E.A. (2017). Xenogenous collagen matrix and/or enamel matrix derivative for treatment of localized gingival recessions: A randomized clinical trial. Part I: Clinical outcomes. J. Periodontol..

[46] Gruber R., Karreth F., Kandler B., Fuerst G., Rot A., Fischer M.B., Watzek G. (2004). Platelet-released supernatants increase migration and proliferation, and decrease osteogenic differentiation of bone marrow-derived mesenchymal progenitor cells under in vitro conditions. Platelets.

[47] Mozgan E.-M., Edelmayer M., Janjić K., Pensch M., Fischer M.B., Moritz A., Agis H. (2017). Release kinetics and mitogenic capacity of collagen barrier membranes supplemented with secretome of activated platelets—The in vitro response of fibroblasts of the periodontal ligament and the gingiva. BMC Oral Health.

[48] Stähli A., Miron R.J., Bosshardt D.D., Sculean A., Gruber R. (2016). Collagen membranes adsorb the transforming growth factor-β receptor I kinase-dependent activity of enamel matrix derivative. J. Periodontol..

[49] Aroca S., Molnár B., Windisch P., Gera I., Salvi G.E., Nikolidakis D., Sculean A. (2013). Treatment of multiple adjacent Miller class I and II gingival recessions with a Modified Coronally Advanced Tunnel (MCAT) technique and a collagen matrix or palatal connective tissue graft: A randomized, controlled clinical trial. J. Clin. Periodontol..

[50] Enriquez-Ochoa D., Robles-Ovalle P., Mayolo-Deloisa K., Brunck M.E.G. (2020). Immobilization of Growth Factors for Cell Therapy Manufacturing. Front. Bioeng. Biotechnol..

[51] Suzuki K., Okada H., Takemura G., Takada C., Tomita H., Yano H., Muraki I., Zaikokuji R., Kuroda A., Fukuda H. (2020). Recombinant thrombomodulin protects against LPS-induced acute respiratory distress syndrome via preservation of pulmonary endothelial glycocalyx. Br. J. Pharmacol..

[52] Linard C., Tissedre F., Busson E., Holler V., Leclerc T., Strup-Perrot C., Couty L., L’Homme B., Benderitter M., Lafont A. (2015). Therapeutic potential of gingival fibroblasts for cutaneous radiation syndrome: Comparison to bone marrow-mesenchymal stem cell grafts. Stem Cells Dev..

[53] Zhang S., Qiu X., Zhang Y., Fu K., Zhao X., Wu J., Hu Y., Zhu W., Guo H. (2015). Basic Fibroblast Growth Factor Ameliorates Endothelial Dysfunction in Radiation-Induced Bladder Injury. BioMed Res. Int..

[54] Pai V.C., Lo I.C., Huang Y.W., Tsai I.C., Cheng H.P., Shi G.Y., Wu H.L., Jiang M.J. (2018). The chondroitin sulfate moiety mediates thrombomodulin-enhanced adhesion and migration of vascular smooth muscle cells. J. Biomed. Sci..

[55] Lo I.C., Lin T.M., Chou L.H., Liu S.L., Wu L.W., Shi G.Y., Wu H.L., Jiang M.J. (2009). Ets-1 mediates platelet-derived growth factor-BB-induced thrombomodulin expression in human vascular smooth muscle cells. Cardiovasc. Res..

[56] Kaneko T., Fujii S., Matsumoto A., Goto D., Ishimori N., Watano K., Furumoto T., Sugawara T., Sobel B.E., Kitabatake A. (2002). Induction of plasminogen activator inhibitor-1 in endothelial cells by basic fibroblast growth factor and its modulation by fibric acid. Arterioscler. Thromb. Vasc. Biol..

[57] Jin H., Choung H.W., Lim K.T., Jin B., Jin C., Chung J.H., Choung P.H. (2015). Recombinant Human Plasminogen Activator Inhibitor-1 Promotes Cementogenic Differentiation of Human Periodontal Ligament Stem Cells. Tissue Eng. Part A.

[58] Aburima A., Berger M., Spurgeon B.E.J., Webb B.A., Wraith K.S., Febbraio M., Poole A.W., Naseem K.M. (2021). Thrombospondin-1 promotes hemostasis through modulation of cAMP signaling in blood platelets. Blood.

[59] Tan K., Lawler J. (2009). The interaction of Thrombospondins with extracellular matrix proteins. J. Cell Commun. Signal..

[60] Horiguchi H., Jin L., Ruebel K.H., Scheithauer B.W., Lloyd R.V. (2004). Regulation of VEGF-A, VEGFR-I, thrombospondin-1, -2, and -3 expression in a human pituitary cell line (HP75) by TGFbeta1, bFGF, and EGF. Endocrine.

[61] Breitkopf K., Sawitza I., Westhoff J.H., Wickert L., Dooley S., Gressner A.M. (2005). Thrombospondin 1 acts as a strong promoter of transforming growth factor beta effects via two distinct mechanisms in hepatic stellate cells. Gut.

[62] Murphy-Ullrich J.E., Poczatek M. (2000). Activation of latent TGF-beta by thrombospondin-1: Mechanisms and physiology. Cytokine Growth Factor Rev..

[63] Belotti D., Capelli C., Resovi A., Introna M., Taraboletti G. (2016). Thrombospondin-1 promotes mesenchymal stromal cell functions via TGFβ and in cooperation with PDGF. Matrix Biol..

[64] Kassem M.M., Helkin A., Maier K.G., Gahtan V. (2019). Thrombospondins Differentially Regulate Proteins Involved in Arterial Remodeling. Physiol. Res..

[65] Agis H., Bauer M., Knebl G., Watzek G., Gruber R. (2008). Effects of platelet-derived growth factor isoforms on plasminogen activation by periodontal ligament and gingival fibroblasts. J. Periodontal. Res..

[66] Patil V.A., Masters K.S. (2020). Engineered Collagen Matrices. Bioengineering.

[67] Gurbuz I., Ferralli J., Roloff T., Chiquet-Ehrismann R., Asparuhova M.B. (2014). SAP domain-dependent Mkl1 signaling stimulates proliferation and cell migration by induction of a distinct gene set indicative of poor prognosis in breast cancer patients. Mol. Cancer.

[68] Parisi L., Buser D., Chappuis V., Asparuhova M.B. (2020). Cellular responses to deproteinized bovine bone mineral biofunctionalized with bone-conditioned medium. Clin. Oral Investig..

